# Should dietary restrictions be imposed on Alzheimer’s Disease patients affected by type 2 diabetes?

**DOI:** 10.1080/19585969.2024.2392491

**Published:** 2024-08-16

**Authors:** Cristina Ștefănescu, Michael Davidson

**Affiliations:** a Department of Dentistry, Faculty of Medicine and Pharmacy, ”Dunărea de Jos” University, Galați, România, Galați, Romania; b Department of Basic and Clinical Sciences, Psychiatry University of Nicosia Medical School, Nicosia, Cyprus

**Keywords:** antidiabetic drugs, type 2 diabetes, Alzheimer disease, vascular dementia patients

## Abstract

**Introduction:**

Antidiabetic drugs, reduction of carbohydrates intake, maintaining normal weight and physical activity are the cornerstone of diabetes 2 treatment.

**Methods:**

This opinion article is not intended to challenge hundreds of studies unequivocally demonstrating the benefits of a healthy lifestyle including appropriate diet in controlling the consequences of T2DM. The article questions whether the benefits of dietary restrictions for the management of T2D in older adults who are already demented, are worth the potential detrimental effects on quality of life for the patients and their caregivers, as well as the effects of dietary restrictions on frailty, sarcopenia.

**Discussion:**

However, the benefit of dietary restrictions including carbohydrates restrictions, might not manifest in elderly Alzheimer and vascular dementia patients with type 2 diabetes. On the contrary, such restrictions might hinder the patients’ and caregiver’s quality of life and encumber attempts to maintain normal weight in a population which tends to be underweight. Therefore, the benefit/risk ratio of dietary restriction should be weighed in this population on an individual basis.

The comorbidity of dementia in general and of Alzheimer Disease (AD)in particular with Type 2 diabetes (T2D) is on the rise (Srikanth et al. 2020) with several factors contributing to this phenomenon. First, both AD and T2D are age-dependent conditions and as longevity increases so does the prevalence of this comorbidity (Saeedi et al. 2019; Sinclair et al. 2000). Second, the two conditions share common risk factors such as metabolic syndrome, and obesity, two conditions which are also on the rise (James et al. 2004). Third, there are pathophysiologic links between the two conditions, such as insulin resistance, inflammation, oxidative stress, and vascular disease, each affecting the prevalence of the other (Dolan et al. 2020). It is estimated that people with diabetes have a X 1.5-2-fold greater risk for AD and vascular subtype. Depending on the population studied and the cut-offs to diagnose the two conditions, about 500 million individuals worldwide suffer from dementia of which about 150 million also suffer from T2D and the respective complications (Bunn et al. 2014; National Diabetes Statistics Report 2017). It is therefore not surprising that this comorbidity has led to the creation of a new syndrome called Diabetes Type 3 (Michailidis et al. 2022). The most frequent complication of T2DM are vascular lesions leading to organ lesions (heart, kidney, brain, skin, retina) (Gavina et al. 2022; Sasako 2023) and occasionally diabetic hyperosmolar coma and diabetic ketoacidosis (Elendu et al. 2023). The risk for complications can be reduced by maintaining the blood glucose levels within certain limits, as well as treating other comorbidities and cardiorenal protective agents.

This opinion article is not intended to challenge hundreds of studies unequivocally demonstrating the benefits of a healthy lifestyle including appropriate diet in controlling the consequences of T2DM. The article questions whether the benefits of dietary restrictions for the management of T2D in older adults who are already demented, are worth the potential detrimental effects on quality of life for the patients and their caregivers,as well as the effects of dietary restrictions on frailty, sarcopenia.

While pre-T2D and mild T2D can be controlled with the help of carbohydrate and fat restrictions, weight loss and physical activity, the cornerstone of T2D management is the combination of antidiabetic drugs with lifestyle changes measured by HbA1C blood levels. However, the range of glycemic control and the optimal mode to achieve these ranges in older adults is still debated (Forouhi 2023) as reflected by the discrepancies between guidelines published in the professional literature (Munshi et al. 2020). While the recommendation for the general population is to maintain HbA1C levels lower than 7·0, the recommendation for older adults with dementia is between 8·0–8·5% (64–69 mmol/mol) (LeRoith et al. 2019; Bellary et al. 2021). However, HbA1C levels may be misleading in older individuals due to anaemia, renal failure, and co-medication. HbA1C is also limited by the fact that it does not capture time point variability but represents an average. Thus, an older person that experiences frequent hypo and/or hyperglycaemia may have the same HbA1C as a person who has good glucose control. While the aim of treatment should be to avoid very high glucose levels (<250 mg/ml), there are several reasons why diabetes control can be more relaxed with higher glucose levels in older adults who are already demented.

*First*, elderly are particularly prone to antidiabetic drug-induced hypoglycaemia because of the impaired sympathetic response (Hoffman 2007). In this already vulnerable population hypoglycaemia, has immediate consequences such as confusion, falls, loss of consciousness, arrhythmias and even death. While still under investigation, events of hypoglycaemia increase the risk and affect the course of dementia (Thorpe et al. 2015; Gómez‐Guijarro et al. 2023). To make matters worse, because of their cognitive and judgement impairment demented individuals may not be able to identify, understand and communicate the hypoglycaemia signs and symptoms and to seek immediate help. *Second*, the accumulation of vascular lesions which characterise T2D generally start before the onset of dementia and, continue until death. However, because the life span of AD and vascular dementia individuals is shorten by the dementia itself, the shorter lifespan also reduces the time frame for the vascular damage to manifest (Lee et al. 2013). *Third*, despite administration of high caloric protein-based food supplements, a common comorbidity of dementia is weight loss (Albanese et al. 2013; Chapman et al. 2021). Furthermore, novel drugs for the treatment of T2D such as semaglutide independently cause weight decrease. Hence, dietary restrictions might hinder efforts to maintain optimal weight.

*Finaly*, there are even more cogent reasons why dietary restrictions should be kept to an essential minimum. Despite the best intentions, and even when sufficient care resources are available, the quality of life of patients suffering from dementia is far from ideal (O'Rourke et al. 2015). Frightening delusions and hallucinations, unprovoked agitation and verbal or physical violence towards the caregivers are only some of the contributors to the poor quality of life. This is true in the best nursing home or at home with the most devoted caregiver. Like non-demented individuals, some of the demented individuals are fond of sweets which may constitute one of the few joys of their life. When sweets products are in sight it is not unusual that a demented individual would “grab” the sweet, the caregiver would try to prevent it, and the event might develop into an argument with the patient becoming agitated adding to the poor quality of life. Furthermore, too often, agitation in dementia is treated with antipsychotic drugs, which, among their adverse effects, may worsen the T2D and elevate glucose levels. However, despite accumulating evidence to the contrary, elderly individuals, particularly in nursing homes, are overtreated for T2D (Deakin and Littley 2001; Sjöblom et al. 2008; Boyle et al. 2013; Sinclair et al. 2018). This opinion manuscript is in no way a call to ignore diet in the management of T2D in the elderly demented but, to raise the dilemmas associated with it. It should not be ignored that chronic hyperglycaemia *via* advanced glycation end products has proinflammatory effects and might damage CNS tissue (Wątroba et al. 2023). Because the dilemma of how to best manage diabetes in elderly demented surfaces often in daily clinical practice, professional organisations of geriatricians, psych-geriatricians nursing home physicians and nurses and diabetologists should consider to provide guidelines specific for this population.
